# Are Isolated Indigenous Populations Headed toward Extinction?

**DOI:** 10.1371/journal.pone.0150987

**Published:** 2016-03-08

**Authors:** Robert S. Walker, Dylan C. Kesler, Kim R. Hill

**Affiliations:** 1 Department of Anthropology, University of Missouri, Columbia, Missouri, United States of America; 2 The Institute for Bird Populations, Point Reyes Station, California, United States of America; 3 School of Human Evolution and Social Change, and Institute of Human Origins, Arizona State University, Tempe, Arizona, United States of America; University of Illinois at Urbana-Champaign, UNITED STATES

## Abstract

At least 50 indigenous groups spread across lowland South America remain isolated and have only intermittent and mostly hostile interactions with the outside world. Except in emergency situations, the current policy of governments in Brazil, Colombia, and Peru towards isolated tribes is a “leave them alone” strategy, in which isolated groups are left uncontacted. However, these no-contact policies are based on the assumption that isolated populations are healthy and capable of persisting in the face of mounting external threats, and that they can maintain population viability in the long-term. Here, we test this assumption by tracking the sizes and movements of cleared horticultural areas made by 8 isolated groups over the last 10–14 years. We used deforestation data derived from remote sensing Landsat satellite sensors to identify clearings, and those were then validated and assessed with high-resolution imagery. We found only a single example of a relatively large and growing population (*c*. 50 cleared ha and 400 people), whereas all of the other 7 groups exhibited much smaller villages and gardens with no sizable growth through time. These results indicated that the smaller groups are critically endangered, and it prompts an urgent re-thinking of policies toward isolated populations, including plans for well-organized contacts that may help save lives and rescue isolated indigenous populations from imminent extinction.

## Introduction

Colonization of the Americas by Europeans brought disease epidemics, slavery, and violence that had catastrophic effects on indigenous populations [1–6]. Despite enormous external pressures over the last 500 years, Greater Amazonia harbors a number of remnant indigenous societies who persist in relative isolation from the outside world [7–10]. The United Nations, national governments, and many non-governmental organizations promulgate no-contact policies for these isolated indigenous populations, with the belief that these people are safest if left to themselves. The no-contact policies assume that currently isolated populations are viable in the long term, and that sustained peaceful contacts would be a greater threat to their health and safety than are the ongoing intermittent, and often hostile, contacts with outsiders [11,12].

The safety and stability of isolated indigenous populations remains largely unknown, and the effectiveness of current no-contact policies is untested [13]. We suggest that policy towards currently isolated populations should be more flexible and contingent upon whether or not each population is growing (Table 1). If populations are small and declining, or not growing as a result of external threats, then current policy approaches should be deemed ineffective. Further, we advocate for contact if it is conducted by well-funded and well-organized governmental agencies which can provide continuous medical care for long periods of time [11]. If a well-organized contact is not feasible, or if isolated people simply refuse peaceful contact, then protection efforts must be bolstered to protect indigenous lands. Regardless, an effective monitoring system is the first necessary step to track the sizes of estimated populations through time and assess the demographic health of isolated indigenous populations, and their genetic and demographic connectivity with other isolated groups, before disease epidemics and other external threats lead to severe depopulation or extinction.

**Table 1:** Suggested plans of action for isolated populations under 4 scenarios.

	Population growing	Population not growing
Future contact well-organized	Maintain isolation and remote surveillance	Contact recommended
Future contact poorly-organized	Maintain isolation and remote surveillance	Improve protection and contact plan

Policy towards currently isolated populations should be contingent on whether or not the current population is growing (columns) and the prospects for whether or not a future contact will be well-organized (rows).

Information on isolated populations historically has been limited to either confirmed reports from aircraft over-flights and encounters on the ground, or unconfirmed reports from potential sightings and material evidence [9,10]. The majority of both isolated and contacted Amazonian indigenous populations live in semi-sedentary villages and practice slash-and-burn horticulture, and for these groups we previously have used remote sensing to measure houses, villages, and gardens to estimate population sizes [14–16]. Even 30 m resolution Landsat and 1 km resolution Moderate Resolution Imaging Spectroradiometer (MODIS) satellite imagery, now compiled into publically-available products, can track deforestation [17] and active fires [18], respectively, like those used to clear villages and gardens. Here, we use these products to identify forest clearings, which were also assessed and validated with high-resolution imagery. Annual clearing rates were then evaluated across time to derive an index of land use changes and agricultural activities in isolated Amazon populations over the last 10–14 years.

## Results

**Fig 1:**
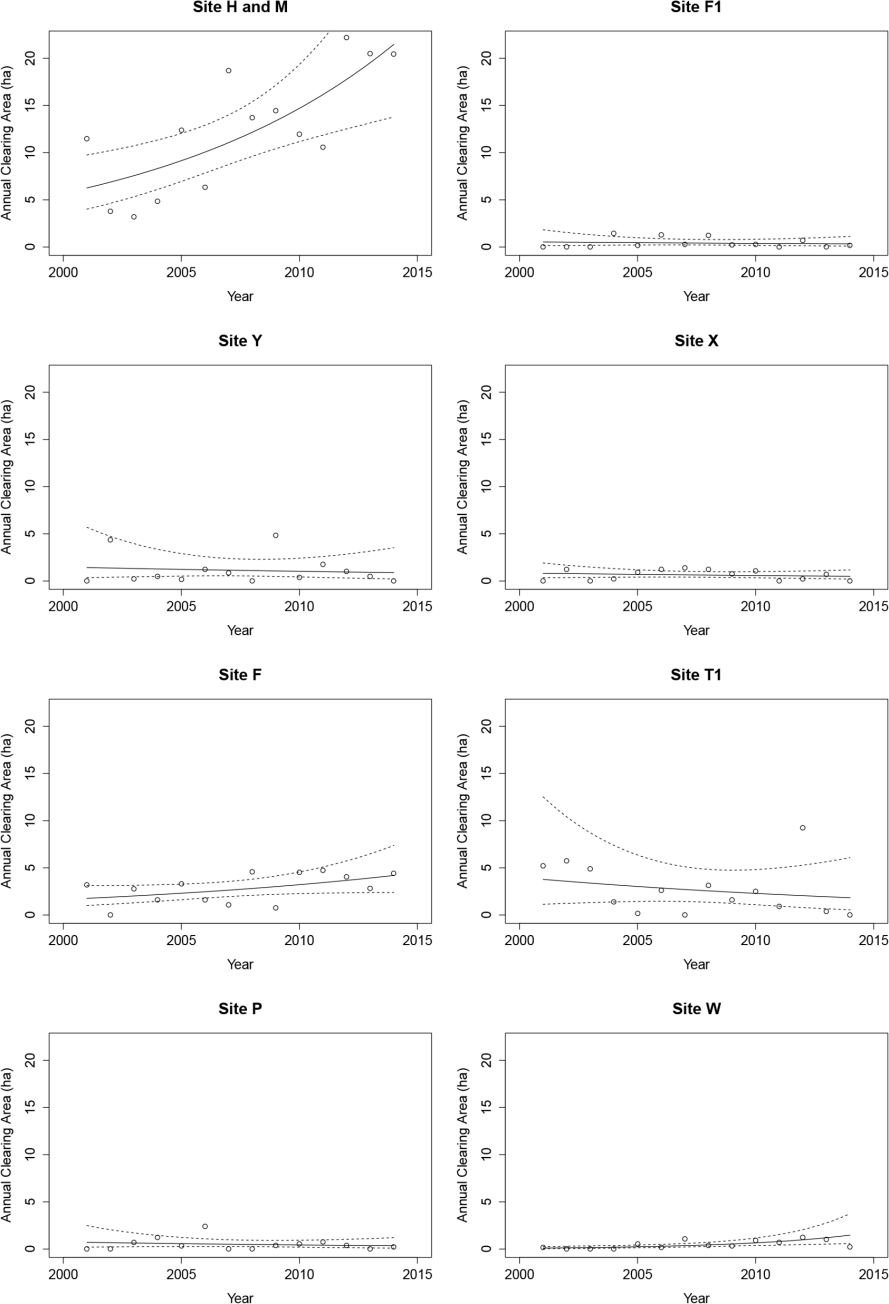
Estimated yearly amount of cleared area from Global Forest Change for each site.

Remote sensing offers the opportunity to track isolated populations through time. Our satellite imagery analysis indicates only a single example of a relatively large and growing metapopulation (*c*. 50 cleared ha and 400 people), whereas all of the other 7 groups appear critically endangered with much smaller villages and gardens, and no sizable growth over time (Fig 1). These other villages are small in terms of total cleared area of villages and gardens (range 2.6–13.3 ha), yielding population estimates of less than 120 individuals (9 people per ha is a rough density estimate for isolated villagers [15]).

Global Forest Change areas (based on Landsat 8 imagery) are converted to 3 year cumulative estimates (see Materials and Methods) that are comparable to high-resolution imagery for particular years where both are available. Direct comparisons between high-resolution satellite imagery and Global Forest Change [17] data show that the latter underestimate total clearing sizes by a mean of approximately 14% (Table 2). Other small cleared areas may not have registered in Global Forest Change algorithms, potentially because they were cleared during the rainy season when clouds obscure them from satellites. Nonetheless, we anticipate that such omissions would have been relatively constant over time and across the study area. Thus, being relatively minor we do not believe the errors change our key result–that only one group is relatively large and growing and all of the others are not. Since our analyses were based on an index of cleared area, constant upward or downward biases in the amount of cleared area detected by the Global Forest Change project would have only a minimal effect on our conclusions.

**Table 2:** Information for the 8 study sites with the year we started to track each site (the end year is 2014).

Site	Ethnicity	Region	Year start	Global Forest Change (ha)	Hi-res (ha)	Error (%)	Migration	Meta-pop.	Status
H+M	Yaminawa	Acre, Brazil	2001	53.3	54.7	2.6	Limited	Yes	vulnerable
F1	Mastanawa	Peru / Brazil	2004	2.8	3.4	16.7	Limited	No	critical
X	Txapanawa	Acre, Brazil	2002	3.2	3.8	15.8	Yes	No	critical
Y	Yanomamö	Roraima, Brazil	2002	8.0	10.5	24.0	Twice	No	critical
F	Flecheiros	Javari, Brazil	2001	13.3	?	?	Limited	Yes	critical
T1	Tsohom Djapá	Javari, Brazil	2001	4.2	4.9	13.9	Limited	Yes	critical
P	Carabayo	SE Colombia	2003	6.0	6.8	11.9	2 Y, 3 N	Yes	critical
W	Waorani	E Ecuador	2001	2.6	3.0	13.3	Limited	Yes	critical
Median			2002	5.1	4.9	13.9	Limited	Yes	critical

Global Forest Change areas here are cumulative estimates that are comparable to high-resolution imagery for particular years where both are available. These are 3 year cumulative areas with the exception of Site P, where a running cumulative is used since they stay in the exact same locations (although 2 were apparently abandoned). Error is the percent difference between Global Forest Change and high-resolution estimates (high-resolution estimates are always larger). Migration describes movement over the study period, and metapopulation the existence or not of multiple villages.

Results from model fitting indicated that 2 of the 8 isolated groups had increasing horticultural capacity across the course of the study period, but only the increase for the group at Site H and M was substantial. This group cleared a mean of 11.6 ha (95% CI 9.2 to 14.6) in each year across the study period with a significantly positive slope (*t* = 3.21; *P* = 0.008), indicating an increasing or accelerating rate of clearing. The group on Site W cleared a small mean level of 0.39 ha per year (95% CI 0.24 to 0.65), and results indicated an annual mean increase in clearing size (*t =* 3.24, *P* = 0.007), but its overall size remains one of the smallest on record. Results for all other groups indicated that there were no discernable trends with regard to changes in clearing sizes through time, and annual clearing amounts were small for each (range 0.40–2.63 ha). Pearson’s goodness of fit statistics indicated no lack of fit for any of the models.

Using MODIS data, we detected 44 fire events, but only within the large Site H and M. All fire detected by the MODIS sensor were within 1 km (approximate resolution of data) of forest clearings identified in Global Forest Change [17], and 30 of the fire detections were within 1 km of patches that were cleared during the same year. Fires were detected between 10:15 h and 14:01 h local time, and dates ranged during the late dry season from 31 July through 17 October, with the bulk occurring in August (*n* = 10) and September (*n* = 28). Of these 44 fires, only 7 occurred prior to 2007 and the other 37 were in 2007 or later with the most fires (*n* = 10) recorded in 2014, consistent with the growth seen in cleared areas through time. No fire signatures were detected for any of the other sites. We interpret this as independent verification that Site H and M is the only large and growing metapopulation, and that all of the other sites have small clearings that, when burned, were too small to be registered by MODIS.

## Discussion

Our results indicated that remote sensing products used to detect forest clearings and fire events are useful tools for evaluating the distributions of indigenous populations living in isolation, and their horticultural activities. Further, results indicated that data derived from remote sensing provided a reliable index of how horticultural capacities changed across time and space, and Global Forest Change provided results comparable to those derived from visual inspections of high-resolution satellite imagery. We detected substantial growth in horticultural capacities of only one group (Site H and M) evaluated herein. Incidentally, this also is the only location where fires were registered by MODIS, and the only location to our knowledge where residents actively come out with bows and shoot arrows at overflying airplanes, instead of hiding in fear.

In addition to the area metrics provided above, we observed what appears to be a fissioning occurring between Site H and M, in which one group divided and subsequently became two groups that moved to the southwest of the original location and the other to the northeast over the 14 year observation period. All of the clearings on both sides of Site H and M were included together within our analysis. This fissioning process is yet another indication of demographic growth.

It is possible that some splinter groups may have migrated outside of our study areas and thus led to underestimation of clearings and population sizes. We determined it unlikely that, if this occurred, the numbers of emigrants are of any substantial size, or we would have detected them during our searches through satellite imagery. Alternatively, external pressure may force isolated peoples to forgo some or all horticultural activities and exploit a more nomadic hunter-gatherer existence. A similar process has likely already occurred for a number of lowland South American societies, including the currently isolated Mashco-Piro [19], and arguably a number of Tupi-Guarani speakers like the Aché, Avá-Canoeiro, Guajá, Sirionó, Xeta, and Yuqui [20]. If groups upon which we focused these analyses transitioned away from horticulture, we may have underestimated population sizes. But again, this type of change is only typical for small populations of people and unlikely to drastically affect our overall result.

### Are currently isolated populations viable?

The evidence that most isolated populations are viable in the long-term appears flimsy. Population estimates of indigenous Brazilians in the 20^th^ century, before sustained peaceful contact, were on average over 5 times larger than populations at contact [13], demonstrating the true risk of severe depopulation during phases of intermittent and hostile interactions with the outside world. The primary challenges are a lethal mix of external forces (illegal logging, mining, squatting, poaching, and narcotrafficking) that threaten the livelihood of isolated populations through displacement, violence, and spread of disease [7,8]. Protection efforts to date have simply not been effective in the many areas where colonization is encroaching into the homelands of isolated peoples.

One of our study locations, Site X of Txapanawa, initiated contact with outsiders in June 2014, which in hindsight was predicted by a decline in cleared areas by this group of some 40 people beginning in 2008. This group migrated from Peru into Brazil over the last 15 years, likely to avoid persecution from drug runners. Their future is uncertain, as there are preliminary reports of respiratory infections, but as of now the Brazilian government is boasting that this is the first contact to occur with no casualties. In a more dire case, there was a single isolated village of Yanomamö, Site Y, that was abandoned in late 2014. It has since become overgrown, as evidenced by satellite imagery and recent over-flights. The current location of these individuals is unknown, which is troublesome given that gold-mining camps are known to be nearby. Isolated, lone villages in sites F1, X, and Y all are generally considered to be in extremely precarious situations, given that they are disconnected from historic metapopulations.

Demographic ties across populations were likely much more robust and frequent before recent development and deforestation fragmented the Amazon Basin systems, and before remaining isolated groups lost connections to neighboring societies. Studies of island species and landscape fragmentation demonstrate that demographic, environmental, and genetic stochasticity make small isolated subpopulations more prone to extinction [21–24]. Metapopulation theory suggests that fringe subpopulations should be more likely to go extinct for a number of reasons—they are small, they are on the edge of usable resource base, they are more isolated (than historic sub-populations at the center of the range) and thus more vulnerable to genetic and demographic disconnect. Fringe populations also are much more likely to lose cultural and technological complexity [25,26].

The only example of a robustly growing metapopulation, that of Site H and M, is welcome. However, the news is not completely positive given that sightings, attacks, and thefts by this group have increased over the last decade [27]. They are sandwiched between 2 contacted indigenous populations, both of whom are pro-contact and living in fear of raids and ransacks [27]. Additionally, settlements and colonization are only some 30 km away in the Brazilian town of Jordão with a new road connecting it to a rubber plantation on another watershed in an area heavily used by isolated peoples [15,27]. As the isolated population grows and contacts with the encroaching outside world increase, the threat of more violence and disease epidemics is expected to worsen.

It is suggestive that Site H and M, which is to our knowledge the only isolated location exhibiting clear signs of population growth, appear to have been in closer proximity around the year 2000 and they have drifted apart in both directions, perhaps indicative of a fission process that commonly characterizes Amazonian villages in growth phase and often associated with violence and tribal warfare [28,29]. It is therefore unclear whether spouses are being exchanged across this divide. We predict that metapopulations of isolated villages, like Site H and M if they are friendly to one another, will show better demographic health than lone isolated villages because of the benefits of being able to exchange marriage partners, goods, and information across communities.

We classify isolated populations with criteria similar to those used by UNESCO to assess the vitality and endangerment of the world’s languages [30]. Key criteria for the endangerment status of isolated populations include population estimates from the sizes of cleared areas, movement and change of cleared areas through time, existence or not of a metapopulation, and distance to external threats (Table 2). For example, an isolated population like that in Site H and M is classified as vulnerable given that they are potentially connected in a metapopulation and have relatively large cleared areas (*c*. 50 ha) and consistent growth. At a higher risk of extinction are all populations at the other 7 locations, with a status set at critically endangered in that our best assessment is that these populations are teetering on extinction. The Yanomamö case mentioned above is a worst case example because they appear to be a lone village disconnected from a metapopulation and have recently migrated to an unknown location (assuming they are not currently extinct).

### Are peaceful contacts devastating?

The common assumption that all contacts with the outside world are devastating is not necessarily true. Of course, this belief does appear consistent with a long history of depopulation driven by epidemics of acute infectious pathogens, or virgin soil epidemics [3,4,31]. European expansion and contact with indigenous populations led to catastrophic depopulation, primarily through the introduction of novel infectious diseases to which native peoples had limited exposure and immunity.

Fortunately, however, a recent ethnohistorical study of indigenous Amazonians shows that mortality rates from contact-related epidemics have declined over the last century [12]. These results highlight the suffering that indigenous populations experienced from an onslaught of infectious diseases and violence introduced by colonization, but also show that the health and contact situation has improved through time. This secular trend in improving survivorship bodes well for future contacts, particularly if they are well-organized and staffed by health care workers. Contact-related mortality can be minimized if the contact team provides both medical treatment and food for sustained periods of time [11,12]. Provided contacted populations do not drop below a minimum of around 30–50 individuals, similar to the current population of Txapanawa, then they have reasonable long-term survival prospects [13].

### Protecting isolated tribes

Returning to suggested plans of action (Table 1), we believe that remote surveillance is a crucial first step to determine whether or not isolated indigenous populations are growing. If populations are growing, as in the case of Site H and M, we can maintain surveillance. However, if populations are not growing and especially if they are small, as in all of the other cases we have studied here, additional action is required to avoid extinction. In fact, the Brazilian government has opted for attempting contact in several recent situations where isolated indigenous populations are tiny, most notably in the case of a small band of Kawahiva in the state of Mato Grosso, and the lone survivor of his tribe “Tanaru” in Rondônia. We advocate for governments to expand their plans for contact provided such efforts are well-organized to deal with the many health challenges that isolated populations face as they come into regular contact with the outside world.

First contacts are difficult endeavors given the remoteness of these areas, challenges in communication, and lack of supplies and health care personnel. However, a well-organized contact can overcome these difficulties with ample resources for a well-trained team that includes translators (often neighboring tribesmen) and doctors. Immediate provisioning of food, shelter, security, antibiotics, vaccines, and antiviral drugs, as well as encouraging isolation of sick and exposed individuals are just some of the basic first steps to mostly eliminate deaths, confusion, and despair that characterized previous contacts. Immediately securing legal land rights and protection for newly contacted groups should also be a priority. Obviously, well-organized contact efforts require considerable funding, qualified personnel, adequate supplies, reliable transportation, and political weight to resist the many powerful economic forces that generally oppose the protection of indigenous peoples.

In sum, the observation that well-organized contacts likely risk fewer lives than under the precarious situations faced by most, if not all, currently isolated populations suggests that the reigning “leave them alone” policy is misguided. Well-organized contacts have the potential to save lives and decrease the risk of ethnolinguistic extinctions. Once a contact occurs, it becomes easier to protect native rights than it otherwise would be for an isolated population. Moreover, in our experiences from interviews with people after contact, there is a unanimous consensus that people stay isolated mostly because of fear of extermination and slavery. People want to trade, particularly for access to steel machetes and axes, and they crave exposure to new ideas and new opportunities. Humans are a gregarious species that intrinsically desire and benefit from outside interactions with other groups. In situations where isolated populations are not currently growing and a well-organized contact is possible, a contact now is recommended given that critically-endangered isolated groups are in desperate need of assistance. A concerted effort is necessary to help save humanity’s last isolated tribes.

## Materials and Methods

**Fig 2:**
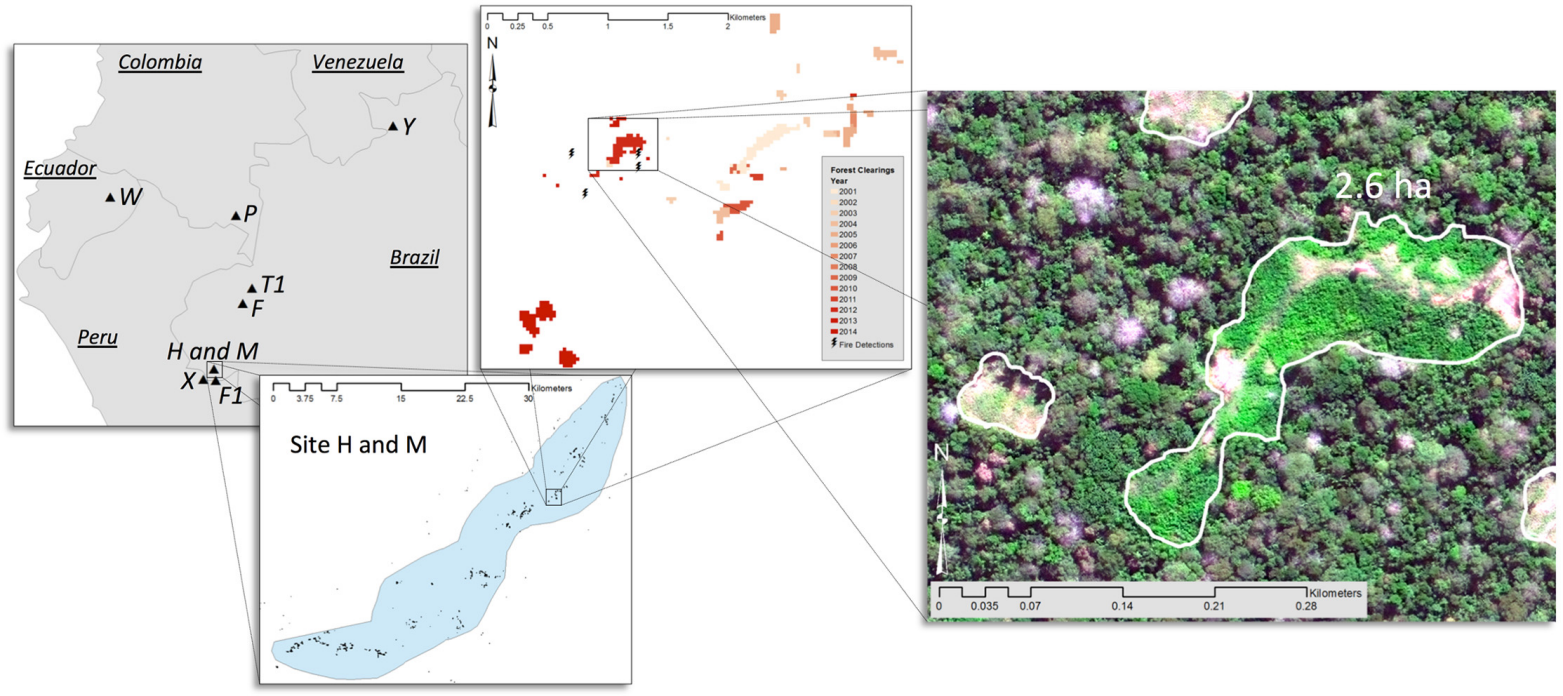
Map of the study sites with inset of Site H and M and a zoom in on a particular village, gardens, and fire detections in Site M. Satellite image by DigitalGlobe.

We scoured freely available high resolution imagery (*c*. 50 cm per pixel, multispectral and panchromatic) from Google Earth (google.com/earth), Flash Earth (flashearth.com), and Terra Server (terraserver.com) to locate the full extents of clearings made by 8 isolated indigenous groups. Most sites are in 2 hotspots near the Peru-Brazil border, along with additional single sites in Colombia, Ecuador, and northern Brazil (Fig 2, Table 2). To augment free imagery of appropriate resolution and timeframe, we purchased commercial imagery for each site from DigitalGlobe, and satellites were tasked to provide up-to-date 2015 imagery for 3 sites (for more imagery information see S1 Table). We truncated data to include only deforestation in each of the 8 sites where the presence of groups of isolated people was previously documented [14–16,32–34]. We used heads-up digitization in ArcMap to create polygons surrounding the total cleared areas of swidden (slash and burn) horticultural fields and village(s).

Hansen and colleagues [17] publish an annually updated geospatial product based on Landsat Enhanced Thematic Mapper Plus archive data that depicts changes in forest cover. It is known as the Global Forest Change project and presents world-wide forest changes used to evaluate deforestation, ranging from small to large scales of analysis [35,36]. The spatial resolution of these measures is *c*. 30 X 30 m per pixel, or 0.09 ha, and we determined that at least 2 contiguous pixels (see below) is suitable to detect the relatively small clearings made by isolated indigenous tribes.

We downloaded forest change digital data from the Global Forest Change project (earthenginepartners.appspot.com) and identified all patches that transitioned from rainforest to clearings in the years between 2001 and 2014 (year of gross forest cover loss–referred in the database as “lossyear”). We encircled each of our 8 sites with polygons, and we used these data to derive an index of the total amount of cleared area in each year. Within each site polygon, we included all clearings detected by Global Forest Change. In total, we identified 806 individual patches that were classified as being deforested over the course of the study period. Visual inspections indicated that single pixel patches were erroneous artifacts in most cases, or perhaps resulting from natural treefalls, and we therefore eliminated 364 single-pixel detections from consideration, yielding a data set comprised of 442 multi-pixel patches. We then amalgamated multiple adjoining deforested patches into 398 contiguous clearings. Of these, 44 forest clearings were combinations of adjoining patches cleared across multiple years (range 2 to 11 years), whereas the remainder represented single-patch clearings that occurred in a single year.

We summed the annual clearing amounts for each of the 8 focal sites, and used those data to represent changes in horticultural capacity between 2001 and 2014. We tested whether garden area for each group grew, shrank, or remained stable across the course of the study. We used program R [37] by fitting linear models to annualized data with a Gamma response distribution and a log-link function. Models were composed of a response variable representing the total amount of area cleared annually at each of the eight sites, an explanatory variable representing year, and an intercept. For each group, we evaluated the parameter estimate for regression slope, and concluded that horticultural capacity was growing across the study period if the parameter estimate was positive and if the 95% confidence interval did not overlap with zero (significance at α = 0.05), as in Site H and M. We concluded that horticultural capacity was not growing if the parameter estimate was not significantly different from zero, as was the case in all other sites. Goodness of fit was assessed using Pearson’s goodness of fit statistics.

We further estimated the total amount of arable horticultural land available to each group by summing areas cleared in Global Forest Change during the previous 3 years. The 3-year production time frame was based on previous research indicating the average time that gardens produce crops for indigenous Amazonians similar to those studied herein [38]. The exception is Site P where we use a running cumulative as they stay in the same locations during the study period. This allowed us to directly compare the amount of cleared area derived from Global Forest Change with the clearing areas measured with heads-up digitization of high-resolution imagery (Table 2, S1 Table).

In a final analysis, we identified dates of forest clearing in each of the focal areas using remote sensing fire detection products from the United States National Air and Space Administration (NASA), which are derived from MODIS and are provided at a 1 km resolution [18]. For each of the focal areas, we downloaded fire detection data from NASA between the years of 2001 and 2015. We then located all forest clearings within 1 km of each fire detection (approximate resolution of MODIS data), and identified whether those fires occurred during the same years as new forest clearings.

## Supporting Information

S1 Table High-resolution imagery information. Here is the information for the recent high-resolution satellite imagery purchased from DigitalGlobe and used for heads-up digitization for each site (PAN = panchromatic, MS = multispectral). We compared the total cleared area with Global Forest Change areas using cumulative estimates over the previous 3 years with the exception of Site P (running cumulative as they stay in the same locations).
(DOCX)
