# Supplementary material for: Are Isolated Indigenous Populations Headed toward Extinction?
Source: PLoS One. 2016 Mar 8;11(3):e0150987. doi: 10.1371/journal.pone.0150987 (PMC4783021; doi:10.1371/journal.pone.0150987)
Supplement: S1 Table — Here is the information for the recent high-resolution satellite imagery purchased from DigitalGlobe and used for heads-up digitization for each site (PAN = panchromatic, MS = multispectral). We compared the total cleared area with Global Forest Change areas using cumulative estimates over the previous 3 years with the exception of Site P (running cumulative as they stay in the same locations). (DOCX) [file pone.0150987.s001.docx]

|  |  |  |  |  |  |  |  |  |
| --- | --- | --- | --- | --- | --- | --- | --- | --- |
| Site | Sensor name | Image date | Spatial resolution (m) | Imaging bands |  |  |  |  |
| H+M | WorldView-03 | Aug 6 2015 | 0.4 | Pan-MS1-MS2 |  |  |  |  |
| F1 | WorldView-02 | Aug 14 2015 | 0.5 | Pan-MS1-MS2 |  |  |  |  |
| X | WorldView-02 | Nov 9 2014 | 0.5 | Pan-MS1 |  |  |  |  |
| Y | WorldView-02 | Jan 8 2010 | 0.5 | Pan-MS1-MS2 |  |  |  |  |
| F | GeoEye-01 | Oct 18 2014 | 0.5 | Pan-MS1 |  |  |  |  |
| T1 | WorldView-01 | Jun 3 2011 | 0.6 | Pan |  |  |  |  |
| P | GeoEye-01 | Jun 5 2015 | 0.6 | Pan-MS1 |  |  |  |  |
| W | WorldView-01 | Aug 6 2012 | 0.6 | Pan-MS1-MS2 |  |  |  |  |

**S1 Table**. Most recent high-resolution satellite imagery purchased from DigitalGlobe and used for heads-up digitization for each site. PAN=panchromatic, MS=multispectral. We compared the total cleared area with Global Forest Change areas using cumulative estimates over the previous 3 years with the exception of Site P (running cumulative as they stay in the same locations).
